# A pragmatic patient-reported outcome strategy for rare disease clinical trials: application of the EORTC item library to myelodysplastic syndromes, chronic myelomonocytic leukemia, and acute myeloid leukemia

**DOI:** 10.1186/s41687-019-0123-4

**Published:** 2019-06-19

**Authors:** Jill A. Bell, Aaron Galaznik, Farrah Pompilus, Sara Strzok, Rafael Bejar, Fatima Scipione, Robert J. Fram, Douglas V. Faller, Stefan Cano, Patrick Marquis

**Affiliations:** 1 Millennium Pharmaceuticals, Inc., (a wholly owned subsidiary of Takeda Pharmaceutical Company Limited), 40 Landsdowne Street, Cambridge, MA 02139 USA; 2 Modus Outcomes, Cambridge, MA USA; 3 UC San Diego Moores Cancer Center – MDS Center of Excellence, La Jolla, CA USA

**Keywords:** Patient-reported outcomes, Quality of life, Myelodysplastic syndromes, Acute myeloid leukemia, Chronic myelomonocytic leukemia

## Abstract

**Background:**

Novel, pragmatic, patient-centered strategies are needed to ensure fit-for-purpose patient-reported outcomes (PRO) instruments in clinical trial research for rare diseases such as myelodysplastic syndromes (MDS), acute myeloid leukemia (AML), and chronic myelomonocytic leukemia (CMML). The objective of the current study was to select supplemental items to add to the European Organization for Research and Treatment of Cancer (EORTC) Quality of Life-Core 30 (QLQ-C30) to ensure content coverage of all important clinical concepts in patients with higher-risk (HR) MDS, low-blast count (LB) AML, and CMML, thus, improving the instrument’s ability to detect clinically meaningful treatment benefit for this context of use.

**Methods:**

Our mixed methods approach comprised literature review, clinician consultation (*n* = 3), and qualitative and quantitative analysis of two stages of patient interview data (*n* = 14, *n* = 18) to select library bank items to supplement a generic cancer PRO, the EORTC QLQ-C30.

**Results:**

Unique symptom (*n* = 54) and impact (*n* = 72) concepts were organized into conceptual frameworks of treatment benefit, compared with EORTC QLQ-C30 items and conceptual gaps identified. Supplemental items (*n* = 13) addressing those gaps were selected from the EORTC Item Library and tested with patients. Supplemental item endorsement frequencies met World Health Organization Quality of Life criteria, suggesting good targeting and relevance for this sample. However, three supplemental items were confirmed as problematic based upon cognitive debriefing results, and expert clinical consultations. Ultimately, 10 supplemental items (*n* = 7 symptom; *n* = 3 impact) were selected for the MDS/AML/CMML context.

**Conclusion:**

Supplemental items were selected to enhance the conceptual coverage of the EORTC QLQ-C30 in the areas of fatigue, shortness of breath, and functioning.

## Introduction

Myelodysplastic syndromes (MDS), acute myeloid leukemia (AML), and chronic myelomonocytic leukemia (CMML) are rare hematological stem cell disorders, associated with anemia, neutropenia, and/or thrombocytopenia, and lead to a variety of symptom and functional impacts. MDS patients fall into five distinct risk categories with an increased likelihood of progressing to AML in the higher-risk (HR) categories [1]. Treatment options for patients with HR MDS include hypomethylating agents, clinical trial treatments, and stem cell transplant [2]. For low-blast count (LB)  AML (which was previously considered refractory anemia with excess blasts in transformation [RAEB-T] and included in the spectrum of HR MDS), treatment strategies include intensive chemotherapy, stem cell transplant, low-intensity chemotherapy, and supportive care [3]. Recommended therapies for CMML generally follow the same guidelines as for higher-risk MDS and AML [2, 3]. Stem cell transplantation is the only potentially curative treatment, but only a small percentage of patients are eligible due to advanced age and co-morbid medical conditions.

Clinicians, researchers, payers, regulatory, and health technology assessment agencies increasingly recognize that patient-reported outcome (PRO) instruments are critical to clinical trials for evaluating the benefits of new treatments on health-related quality of life (HRQOL) and when making treatment decisions [4–7]. However, measuring HRQOL in rare diseases can be challenging, as widely used generic PRO instruments may lack the sensitivity required to demonstrate clinical change brought about by new therapies [8, 9]. A recent Food and Drug Administration (FDA) review of a new cancer treatment [10] may offer a pragmatic solution: the use of existing legacy cancer-specific PRO instruments in conjunction with additional items that are deemed more relevant and important to the specific and current context of use.

When faced with the challenge of measuring the patient experience in the context of MDS, AML, and CMML, we determined that the European Organization for Research and Treatment of Cancer (EORTC) Quality of Life-Core 30 (QLQ-C30), a widely used legacy cancer-specific HRQOL PRO instrument [11], offered promising potential. The EORTC QLQ-C30 has been used in over 3000 studies and has supported labeling claims in the United States (US) and Europe [12, 13]. The EORTC’s Quality of Life Group now offers an Item Library where researchers can select additional items to be used with core questionnaires and disease-specific modules [14]. The Item Library comprises 953 unique items and 67 questionnaires (with some translated in over 100 languages [15]), with conceptually defined scales separating symptoms and impacts. In this study, we developed a pragmatic PRO strategy for supplementing the EORTC QLQ-C30 with the most appropriate additional items to specifically measure treatment benefit for patients with HR MDS, LB AML, and CMML, driven primarily by insights gathered from patients.

## Materials and methods

**Fig. 1:**
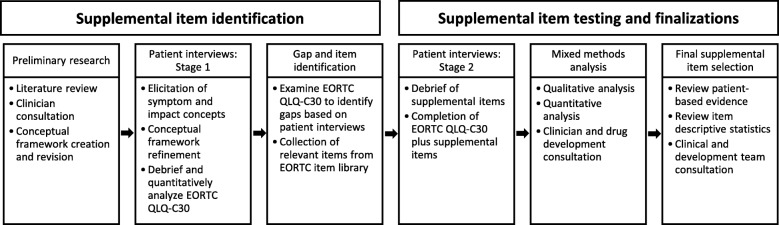
Mixed methods study process

We used a mixed methods approach [16], which included literature review, clinician consultation, qualitative patient interviews, and qualitative and quantitative analysis of patient interview data. This involved the synthesis of qualitative and quantitative data to identify, define, and operationalize PRO instruments as measures of a given concept of interest in a specific context of use. There were two stages: 1) identification of supplemental symptom and impact items; and 2) supplemental item evaluation and finalization. An overview of the study process is provided in Fig. 1.

### Stage 1: supplemental item identification

#### Literature review

We performed a literature review of patient-centered, qualitative studies in MDS and AML published between January 2000 and July 2016 to gain an initial understanding of disease- and treatment-related symptoms and impacts. Patient-identified symptom and impact concepts in MDS and AML were extracted from these studies, compiled, and organized into hypothesized conceptual frameworks of treatment benefit [17–19].

#### Clinician consultation

Three clinicians experienced in treating hematological disorders were individually consulted to gather additional information on the signs, symptoms, impacts, and treatment benefits/risks of MDS, AML and CMML. Clinicians also reviewed the symptoms, impacts, and hypothesized conceptualizations generated from the literature review data and provided suggestions for revising the preliminary frameworks.

#### Stage 1 patient interviews

##### Study sample and interview conduct

After Independent Review Board (IRB) approval of the study protocol (Quorum Review IRB, reference #32211/1), patients were recruited through one of two sources: 1) advertisements posted by the MDS Foundation, Inc. on their patient message board, and 2) physician referrals from three US-based clinical offices. Participating patients provided written informed consent. Eligible patients were ≥ 18 years of age; spoke, read, and understood English; had a diagnosis of HR MDS, LB count AML, or CMML; and had an Eastern Cooperative Oncology Group status of 0–2 [20, 21]. Patients were excluded if they had received an allogenic stem cell transplant or intensive chemotherapy. All one-on-one interviews lasted approximately one hour, were conducted by telephone, audio-recorded, and transcribed.

##### Concept elicitation, cognitive debriefing, and qualitative data collection

Open-ended, semi-structured concept elicitation interviews were performed to better understand the patients’ experience of both the symptoms and impacts of their disease. Patients were debriefed to assess their understanding of the items in the EORTC QLQ-C30. A “think aloud” process was used to confirm item relevance and determine whether the patients interpreted the items and response options in the manner intended [22]. Item responses were collected to enable quantitative analysis of EORTC QLQ-C30 data in this patient population.

##### Qualitative analysis

Concept elicitation transcripts were analyzed thematically [23, 24] using detailed line-by-line coding [25] to examine, compare, and develop treatment benefit concepts using ATLAS.ti software [26]. Conceptual saturation was assessed by ordering interviews chronologically, then grouping interviews into quantiles and comparing concepts emerging by each sequential quantile to assess whether saturation was reached (i.e., no new concepts emerged). Stage 1 symptom and impact concepts were added to the concepts identified through literature review and clinician consultation and data were used to revise the emerging conceptual frameworks of treatment benefit. Cognitive debriefing analysis for EORTC QLQ-30 items was conducted using a coding framework to organize and catalogue patient interpretation, assessment of relevance, and responses to the core instrument.

##### Quantitative analysis

Item-level endorsement frequency analysis was performed to describe the distribution of responses to the items using SPSS 24.0 software. The World Health Organization Quality of Life (WHOQOL) criteria were used for interpreting the results (maximum criterion of < 80% for endorsement frequencies; minimum criterion of > 10% for aggregate endorsement frequencies; in other words the minimum criterion for the sum of two adjacent categories [27]).

#### Gap analysis and supplemental item identification

The symptoms and impacts identified from the literature review, clinician consultation, and Stage 1 patient interviews were compared with the EORTC QLQ-C30 items to identify measurement gaps and to guide the selection of supplemental items from the EORTC Item Library to address the instrument’s conceptual gaps. The following criteria guided supplemental item selection:

- Concept was NOT primarily considered a side effect of treatment
- Concept was strongly endorsed by patients or considered a core symptom/impact by clinicians
- Concept had potential to demonstrate treatment efficacy

### Stage 2: supplemental item testing, final item selection

#### Stage 2 patient interviews

The patient population inclusion/exclusion criteria, cognitive debriefing interview methods, and analysis were the same as for Stage 1 interviews.

#### Final supplemental item selection

Cognitive debriefing interviews were followed by an interview with a clinical expert to review patient feedback and provide clinical insight on items that may assess treatment benefit. Items were further discussed with the drug development team to determine whether the drug’s mechanism of action was likely to impact the identified symptom and impact concepts. The final supplemental items were selected based on evidence generated from patient interviews, the item descriptive statistics, and clinical consultation.

## Results

### Stage 1: supplemental item identification

#### Literature review

Of the 84 studies identified in the initial database search, only four of these proved to be qualitative articles focused on the patient-reported experience of MDS or AML. A total of 31 symptom concepts and 48 impact concepts were identified from these studies. These concepts were organized into draft hypothesized conceptualizations of treatment benefit for MDS and AML patients comprising seven symptom domains and eight impact domains. In Table 2 below, there are 30 symptom concepts from the literature; muscle pain and muscle soreness were collapsed from two separate concepts into one (muscle pain/soreness). In Table 3 below, there are 43 impact concepts from the literature; five concepts (problems walking in certain places, problems walking long distances, problems walking on unleveled ground, problems walking up and down stairs, and unsteady gait) were collapsed into one concept (walking).

#### Clinical consultation

Clinicians reviewed the symptoms and impacts extracted from the literature, highlighted the importance of fatigue, shortness of breath, and the significant impact on patient functioning, and identified additional concepts not found in the literature search. All clinician feedback was considered and incorporated into the emerging symptom and impact conceptualizations, which retained the original hypothesized domains.

#### Stage 1 patient interviews

##### Study sample

The Stage 1 study sample included 14 patients; Stage 2 included 18 patients. All enrolled patients completed the study (see Table 1).

**Table 1:** Patient demographic and clinical characteristics

Sample characteristics		Stage 1 (*n* = 14)	Stage 2 (*n* = 18)
Recruitment source			
MDS Foundation		6 (43%)	11 (61%)
Physician referral		8 (57%)	7 (39%)
Diagnosis type			
Higher-risk MDS		11 (79%)	14 (78%)
Low-blast count AML		1 (7%)	1 (6%)
CMML		2 (14%)	3 (17%)
Age, years			
Mean (SD)		68.8 (±8.48)	68.1 (±10.1)
Minimum		54	50
Maximum		83	83
Gender			
Female		9 (64%)	10 (56%)
Male		5 (36%)	8 (44%)
Education level			
Post-graduate degree		5 (36%)	5 (28%)
Undergraduate degree		1 (7%)	1 (6%)
Some college		3 (21%)	3 (17%)
Trade/technical degree		0 (0%)	1 (6%)
High school/General Educational Development equivalent		4 (29%)	6 (33%)
Some high school or less		1 (7%)	2 (12%)
Employment status			
Retired		8 (57%)	10 (56%)
Part-time		3 (21%)	3 (17%)
Full-time		2 (14%)	1 (6%)
Disability		1 (7%)	3 (17%)
Not employed		0 (0%)	1 (6%)
Eastern Cooperative Oncology Group status			
Clinician-reported	0 – fully active	1 (7%)	1 (6%)
	1 – restricted in physically strenuous activity	7 (50%)	6 (33%)
Patient-reported	1 – difficulty with physically strenuous activity	1 (17%)	5 (28%)
	2 – able to walk and care for self, restricted in work activities	5 (36%)	6 (33%)
Classification (clinician-reported only)		*n* = 8	*n* = 7
FAB- Refractory anemia		1 (13%)	1 (14%)
WHO-RAEB1		3 (38%)	2 (29%)
WHO-RAEB2		1 (13%)	1 (14%)
FAB- Refractory anemia, WHO RAEB1		2 (25%)	2 (29%)
FAB-CMML and WHO-CMML1		1 (12%)	1 (14%)
Prognostic risk category^b^ (MDS only)		*n* = 11	*n* = 10
Very high		4 (36%)	3 (30%)
High		3 (27%)	3 (30%)
Intermediate		2 (18%)	2 (20%)
Unknown		2 (18%)	2 (20%)
Hemoglobin level			
7.0–9.9		6 (43%)	9 (50%)
10.0–11.9		6 (43%)	6 (33%)
12.0–13.0		2 (14%)	3 (17%)
% myeloblasts in bone marrow (patient-reported only)		*n* = 6	*n* = 10
< 1%		1 (17%)	1 (10%)
1–9.9%		3 (50%)	5 (50%)
10–11%		1 (17%)	1 (10%)
Missing data		1 (17%)	3 (30%)
Treatment^a^			
Azacitidine		9 (64%)	12 (67%)
Decitabine		2 (14%)	2 (11%)
G-CSF; copper gluconate; ondansetron; sulfamethoxazole / trimethoprim; levofloxacin; valacyclovir; acetaminophen/hydrocodone; oxycodone; prochlorperazine		1 (7%) each	1 (6%) each
Unidentified clinical trial study drug		0 (0%)	1 (6%)
No treatment		0 (0%)	1 (6%)

*Abbreviations*: *AML* Acute myeloid leukemia, *CMML* Chronic myelomonocytic leukemia, *FAB* French American British, *G-CSF* Granulocyte-colony stimulating factor, *MDS* Myelodysplastic syndromes, *RAEB* Refractory anemia with excess blasts, *SD* Standard deviation, *WHO* World health organization

^a^Not mutually exclusive, missing treatment data for 1 patient

^b^Per International Prognostic Scoring System-Revised [30]

##### Concept elicitation results

Forty-seven disease and treatment-related symptom concepts and 53 disease and treatment-related impact concepts spontaneously arose from patient interviews. All patients experienced fatigue, which was reported by patients as one of the most bothersome symptoms. Patients reported feeling easily fatigued, tired, low energy, and exhaustion. Most patients also reported experiencing shortness of breath, weakness, pain, nausea, bruising, constipation, and dizziness. Disease-related symptoms and side effects of treatments were also reported to have substantial impact on patients’ HRQOL; including difficulty performing daily activities, walking, doing leisure activities, and participating in activities that could expose them to infection (such as eating out, traveling, and caring for others). Concepts from these 14 interviews were analyzed for saturation. The four new codes that emerged during the final quantile did not provide additional information to inform the conceptual framework, therefore saturation was considered achieved. The symptom and impact conceptualizations were updated with the additional symptom and impact data and retained the original hypothesized domains (see Tables 2 and 3).

**Table 2:** Consolidated symptom conceptualization of patient experience with MDS, AML and CMML

Disease- and Treatment-Related Symptoms of Higher-Risk MDS, Low-Blast Count AML, and CMML			
	PT	LIT	CLIN^a^
Gastrointestinal			
Nausea	✓	✓	✓
Constipation	✓	✓	✓
Vomiting	✓	✓	
Diarrhea	✓		✓
Distension	✓		
Feeling full after eating little food	✓		
Bloating	✓		
Loss of appetite	✓		
Fatigue			
Weakness	✓	✓	✓
Easily fatigued	✓	✓	✓
Tiredness	✓	✓	✓
Low Energy	✓	✓	✓
Exhausted	✓		
Heaviness (arms/legs)	✓	✓	
Sluggish	✓	✓	✓
Worn out	✓		✓
Pain			
Bone pain	✓	✓	
Muscle pain/soreness	✓	✓	✓
Headache	✓	✓	✓
Cramps	✓		
Chemical burn	✓		
Bleeding			
Bleeding	✓	✓	✓
Bruising	✓		✓
Petechiae	✓		
Cognitive function			
Difficulty concentrating	✓	✓	✓
Memory loss	✓		
Confusion	✓		
Sensory			
Dry throat/mouth	✓	✓	
Tastelessness		✓	
Taste changes	✓		
Dry lips		✓	
Ringing in the ears		✓	
Numbness		✓	✓
Cold hands/feet		✓	
Feeling cold	✓		
Itchy skin	✓		
Other			
Shortness of breath	✓	✓	✓
Rash	✓		✓
Infection (e.g., cough, chest congestion, sputum production, dysuria)	✓		✓
Dizziness	✓	✓	
Lightheaded	✓		
Dyspnea on exertion	✓	✓	✓
High fever/chills	✓	✓	✓
Paleness	✓	✓	
Weight gain/loss	✓	✓	
Hair loss	✓	✓	
Vertigo	✓	✓	
Anorexia		✓	✓
Cardiac issues	✓		✓
Eyes tearing	✓		
Skin peeling	✓		
Skin tearing	✓		
Swelling (lymph nodes)	✓		
Dysphagia (difficulty swallowing)		✓	

✓ indicates concept explicitly endorsed by referenced source

*Abbreviations*: *PT* Patient-reported concept, *LIT* Literature-based concept, *CLIN* Clinician-supported concept

^a^Clinicians reviewed and endorsed all literature-based concepts in this framework

**Table 3:** Consolidated impact conceptualization of patient experience with MDS, AML and CMML

Health-Related Quality of Life Impacts of Higher-Risk MDS, Low-Blast Count AML, and CMML			
	PT	LIT	CLIN^a^
Mobility			
Walking (e.g., problems walking long distances, walking in certain places, walking on unleveled ground, walking on stairs, unsteady gait)	✓	✓	✓
Stay in bed/chair	✓		
Inability to move quickly (move slowly)	✓	✓	
Exercising	✓	✓	
Loss of coordination	✓		
Rising from sitting	✓		
Bending down	✓		
Lack of balance	✓		
Falling	✓		
Standing	✓		
Sleep			
Insomnia	✓	✓	
Feeling sleepy	✓	✓	✓
Waking from sleep	✓	✓	
Sleep disturbances	✓	✓	
Work/Finances			
Inability to carry out jobs	✓	✓	✓
Time lost from work	✓	✓	
Loss of employment	✓	✓	
Treatment costs	✓	✓	
Leisure			
Recreational activities (e.g., bowling, golfing, sporting events, fishing, bird watching)	✓	✓	✓
Yardwork	✓		
Limited air travel	✓	✓	
Watching television		✓	
Watching movies/theater	✓		
Taking extending vacations		✓	✓
Arts and crafts	✓		
Reading	✓		
Writing	✓		
Board games	✓		
Diet and nutrition			
Avoid dining out	✓		
Avoid certain foods		✓	
Psychological Impact			
Treatment burden	✓		
Low motivation	✓	✓	
Anxiety		✓	✓
Worry		✓	✓
Feeling discouraged		✓	
Sadness		✓	
Increased sense of awareness	✓		✓
Guilt	✓		
Depression	✓	✓	
Anger	✓	✓	
Frustration	✓	✓	
Irritability		✓	
Loss of confidence	✓		
Distress		✓	
Feeling overwhelmed		✓	
Stress		✓	
Strengthening pre-existing faith		✓	
New-found appreciation		✓	
Struggle to find meaning in one’s illness		✓	
Feeling uncertainty		✓	
Bored		✓	
Fear		✓	✓
Social limitation			
Isolation	✓	✓	✓
Wearing protective masks	✓	✓	
Exposure to/ interaction with children/ grandchildren	✓	✓	✓
Relationships	✓	✓	✓
Sex life	✓		
Attendance at parties/celebrations	✓		
Restricting visits from sick people	✓	✓	✓
Attending church	✓		
Inability to maintain roles		✓	✓
Activities and daily living			
Shopping	✓		
Childcare	✓	✓	
Household chores	✓	✓	✓
Caring for others	✓		✓
Driving	✓		
Grooming	✓		
Showering/bathing	✓		
Need supervision bathing	✓		
Getting dressed	✓		
Traveling to hospital		✓	

✓ indicates concept explicitly endorsed by referenced source

*Abbreviations*: *PT* Patient-reported concept, *LIT* Literature-based concept, *CLIN* Clinician-supported concept

^a^Clinicians reviewed and endorsed all literature-based concepts in this framework

##### EORTC QLQ-C30 cognitive debriefing and item endorsement results

Patients generally found the items of the EORTC QLQ-C30 acceptable and clear. Overall, the endorsement frequencies showed a good spread, indicating that most of the items were relevant to this sample (see Table 4). Some items showed high floor effects, indicating fewer problems with these symptoms/functions in this population; examples included nausea, vomiting, difficulty concentrating, and needing help eating, dressing, and washing.

**Table 4:** EORTC QLQ-C30: Item-level endorsement frequencies (*n* = 14) from Stage 1

EORTC QLQ-C30								
		Not at all %		A little %		Quite a bit %		Very much %
1	Strenuous activities	7.1		28.6		42.9		21.4
2	Long walk	7.1		14.3		42.9		35.7
3	Short walk	35.7		50.0		14.3		0.0
4	Stay in bed/chair	57.1		21.4		14.3		7.1
5	Need help eating, dressing, washing^a^	85.7		14.3		0.0^a^		0.0^a^
6	Limited work or daily activities	14.3		57.1		28.6		0.0
7	Hobbies	35.7		28.6		14.3		21.4
8	Shortness of breath	14.3		64.3		14.3		7.1
9	Pain	50.0		21.4		21.4		7.1
10	Need to rest	7.1		35.7		35.7		21.4
11	Trouble sleeping	42.9		35.7		7.1		14.3
12	Feeling weak	7.1		57.1		14.3		21.4
13	Lack of appetite	28.6		50.0		42.9		7.1
14	Nausea^a^	50.0		42.9		7.1^a^		0.0^a^
15	Vomiting^a^	71.4		28.6		0.0^a^		0.0^a^
16	Constipation	28.6		28.6		28.6		14.3
17	Diarrhea^a^	71.4		21.4		0.0^a^		7.1^a^
18	Tiredness	0.0		50.0		28.6		21.4
19	Pain interfere daily activities	42.9		28.6		14.3		14.3
20	Difficulty concentrating	50.0		35.7		14.3		0.0
21	Feeling tense	35.7		50.0		14.3		0.0
22	Worry	14.3		57.1		21.4		7.1
23	Irritable mood	14.3		71.4		14.3		0.0
24	Depressed mood	42.9		42.9		7.1		7.1
25	Remembering things	42.9		35.7		21.4		0.0
26	Family life	35.7		28.6		21.4		14.3
27	Social activities	14.3		28.6		50.0		7.1
28	Financial difficulties	57.1		28.6		7.1		7.1
		1 Very poor %	2%	3%	4%	5%	6%	7 Excellent %
29	Overall health	0.0	7.1	14.3	14.3	35.7	28.6	0.0
30	Overall quality of life	0.0	0.0	28.6	21.4	21.4	14.3	14.3

^a^Item falls short of the WHOQOL criteria

#### Gap analysis and supplemental item identification

Fifty-four unique symptom concepts and 72 unique impact concepts were identified; 18/54 symptoms and 30/72 impacts arose exclusively from patient interviews. The consolidated frameworks of symptoms and impacts are illustrated in Tables 2 and 3.

We compared symptom and impact concepts elicited from all sources to the items of the EORTC QLQ-C30 and identified conceptual gaps of the instrument in this context of use. Areas for possible measurement improvement due to gaps in the conceptual coverage were highlighted and 13 supplemental items from the EORTC Item Library were selected: bone pain [31], weakness (lack of physical strength, muscle weakness), fatigue (mobility), easily fatigued, lack of energy [32], bruising [14], dizziness/light headedness [28], shortness of breath [14], dyspnea on exertion [31], traveling to medical appointments/general travel [29], household chores [33], shopping/running errands [32]. Of note, one key concept (nosebleeds) met the item inclusion criteria, but it was not in the EORTC Item Library at the time of supplemental item selection and thus not included. An item around nosebleeds has since been added to the EORTC Item library.

### Stage 2: supplemental item testing, final item selection

#### Stage 2 patient interviews

##### Cognitive debriefing results

Most supplemental items were relevant and generally well understood. Some patients attributed the “bone pain” item to age, injury, or arthritis rather than to their disease or treatments. Several patients found the “travel limitations” item unclear, as they were unsure what type of travel to consider (e.g., car travel vs. air travel, long vs. short journey). Finally, patients identified some issues with EORTC supplemental items such as “difficulty with stairs or getting out of a chair due to weakness,” which target more than one concept. For instance, some patients had difficulty with stairs but no trouble getting out of a chair, which made selecting a response option difficult.

##### Item-level endorsement frequency results

All but four of the EORTC QLQ-C30 items met the WHOQOL criteria and showed satisfactory endorsement frequencies with a good spread of responses, indicating that these items are generally relevant and well-targeted to this patient sample (Table 5). All 13 supplemental items met the item level criteria, suggesting good targeting and relevance for this sample.

**Table 5:** EORTC QLQ-C30 and supplemental items: Item-level endorsement frequencies (*n* = 18) from Stage 2

EORTC QLQ-C30								
		Not at all %		A little %		Quite a bit %		Very much %
1	Strenuous activities	0.0		38.9		44.4		16.7
2	Long walk	0.0		22.2		33.3		44.4
3	Short walk	27.8		55.6		11.1		5.6
4	Stay in bed/chair	27.8		44.4		27.8		0.0
5	Need help eating, dressing, washing^a^	77.8		16.7		5.6^a^		0.0^a^
6	Limited work or daily activities	11.1		44.4		33.3		11.1
7	Hobbies	33.3		33.3		27.8		5.6
8	Short of breath	27.8		38.9		22.2		11.1
9	Pain	27.8		16.7		27.8		27.8
10	Need to rest	16.7		27.8		33.3		22.2
11	Trouble sleeping	33.3		22.2		27.8		16.7
12	Felt weak	16.7		38.9		16.7		27.8
13	Lacked appetite	61.1		16.7		16.7		5.6
14	Nauseated	77.8		5.6		11.1		5.6
15	Vomited^a^	88.9^a^		5.6^a^		5.6^a^		0.0^a^
16	Constipated	22.2		33.3		33.3		11.1
17	Diarrhea	66.7		22.2		5.6		5.6
18	Tired	11.1		44.4		22.2		22.2
19	Pain interfere daily activities	33.3		27.8		16.7		22.2
20	Difficulty concentrating	50.0		27.8		22.2		0.0
21	Feel tense^a^	33.3		61.1		5.6^a^		0.0^a^
22	Worry	22.2		61.1		5.6		11.1
23	Irritable	22.2		61.1		11.1		5.6
24	Depressed^a^	33.3		61.1		0.0^a^		5.6^a^
25	Remembering things	44.4		44.4		11.1		0.0
26	Family life	33.3		44.4		11.1		11.1
27	Social activities	22.2		27.8		33.3		16.7
28	Financial difficulties	38.9		38.9		16.7		5.6
		1 Very poor %	2%	3%	4%	5%	6%	7 Excellent %
29	Overall health	0.0	0.0	33.3	27.8	22.2	11.1	5.6
30	Overall quality of life	0.0	0.0	16.7	27.8	22.2	11.1	22.2
Supplemental items								
		Not at all %		A little %		Quite a bit %		Very much %
1	Bone aches or pains	38.9		27.8		16.7		16.7
2	Arms/legs weak	27.8		16.7		33.3		22.2
3	Slowed down	11.1		27.8		44.4		16.7
4	Easily tired	11.1		33.3		38.9		16.7
5	Lacked energy	16.7		22.2		44.4		16.7
6	Bruise	44.4		38.9		11.1		5.6
7	Dizzy	66.7		22.2		0.0		11.1
8	Exertion shortness of breath	22.2		22.2		44.4		11.1
9	Stop for breath when walking	33.3		27.8		27.8		11.1
10	Stairs/getting up from chair	27.8		27.8		27.8		16.7
11	Travel ability limitations	66.7		16.7		16.7		0.0
12	Heavy housework	11.1		16.7		27.8		44.4
13	Shopping exhausting	27.8		11.1		33.3		27.8

^a^Item falls short of the WHOQOL criteria

Patterns of endorsement frequencies suggested patients appeared to have more problems with strenuous activities and fewer problems with staying in bed/chair, vomiting, concentrating, feeling tense, depressed, remembering things, travel limitations, and overall health and quality of life items. Endorsement frequencies of 0% at the two ends of the scale for these items could further inform item relevance and indicate fewer/more problems associated with the symptoms/functions of these items compared to the rest. It is worth noting that test design issues were detected for one EORTC QLQ-C30 item (need help eating, dressing, washing) and one supplemental item (difficulty with stairs or getting up from chair), which target more than one concept.

##### Selection of final supplemental items

Cognitive debriefing of the 13 proposed supplemental items indicated important comprehension issues with two items: bone pain and travel limitations. No concerns were raised for any of the items based on the quantitative analyses. A clinical expert reviewed Stage 2 patient data and identified four supplemental items (bone pain, bruising, dizzy, and travel limitations) as less relevant when considering overall treatment benefit. Finally, consultation with clinical experts and the drug development team indicated that bruising may be associated with treatment administration and therefore unlikely to demonstrate treatment benefit in the clinical trial context as all treatments and supportive care are administered intravenously or via injections. Based upon all the amassed input, three items (bone pain, bruising, and travel limitations) were removed from the supplemental item set leaving seven symptom and three impact items (see supplemental materials).

## Discussion

Given their complexities, rare disease clinical trials require PRO strategies that are flexible and innovative [4]. In our study, integrating data from different sources through a mixed methods framework provided a pragmatic and efficient approach to maximizing the applicability of a legacy PRO instrument in a new context of use [15]. The initial literature review, consultation with clinicians, and interviews with patients led to an improved conceptual framework, thus enabling us to select and test supplemental items from the EORTC Item Library relevant to the HR MDS, LB AML and CMML context that addressed concepts that were not captured by the EORTC QLQ-C30. We believe this study illustrates a promising method for selecting supplemental items from the EORTC Item Library to capture specific concepts not covered in the EORTC QLQ-C30 for use in therapeutic trials in different cancer contexts.

Our work began with this same emphasis on understanding the patients’ perspectives of the symptoms and impacts of their disease. The literature review highlighted the dearth of patient-focused, qualitative research in the targeted conditions. Our work with patients contributes to the literature on the patient experience of these diseases, particularly as 18 of the symptoms and 30 of the impacts identified from patient interviews arose exclusively from patients and were not identified in earlier research. This information was combined with perspectives from health care professionals, researchers, and all other patient-based evidence available to illustrate relationships among the most important signs, symptoms, concerns, and disease impacts. In the rare disease context, sample sizes will always be small, so it is imperative to pay careful attention to the patient voice. In these situations, combining fidelity to the patient voice with small scale quantitative analyses and re-testing iterations with patients is a pragmatic approach to instrument choice and development.

A key strength of this research is its broad evidence base and incorporation of findings from all stakeholders, which, particularly in the rare disease context, can lead to a consensus on the best way to collect and report key outcomes [4], while still placing the patient’s voice at the center of measurement. Consultation with clinicians and drug development researchers at several stages of the project provided a practical perspective on which patient-identified symptoms and impacts were likely to show treatment benefit in the specific clinical trial under consideration – this approach can be generalized to other concepts important to patients in this and other contexts of use.

For example, in a previous project that aimed to improve targeting of the 12-item Multiple Sclerosis Walking Scale for higher functioning multiple sclerosis patients, a Gait Module was developed through a multi-phase mixed method study design that included concept elicitation, item generation, cognitive debriefing, and Rasch analysis [34]. Supplemental items were also “bolted-on” to the ABILHAND, a PRO instrument designed to assess manual ability, by employing a mixed methods approach to enhance its sensitivity to change and reduce ceiling effects [35]. Both studies were based on a thorough understanding of the patients’ perspectives on their disease and a thoughtful conceptualization of treatment benefit using information from both clinical experts and published literature as the foundation for selecting items to expand the measurement range of the existing instruments. We hope these sorts of studies will be the beginning of a growing body of research.

Limitations of this work should be acknowledged. The initial conceptualization of treatment benefit did not include CMML patients; however, this patient perspective was addressed during the patient interview phases of the study. As is typical in rare disease studies, the sample size was small (though representative of the patients with these conditions) and some patients were recruited through support/advocacy groups; both of these factors could potentially limit the generalizability of our findings.

Furthermore, only about half of the patients from each stage had a clinically confirmed diagnosis. Demographic questions gathered information about patient characteristics that helped provide supporting evidence of their diagnosis; additionally, a small-scale analysis indicated no significant differences between data collected from patients with confirmed vs. non-confirmed diagnoses. Finally, few patients were managed with supportive care only, which offered challenges in terms of understanding the burden of disease pertaining to symptoms and impacts versus those related to treatment, though this was carefully considered in our literature review and clinician consultations. Given the limitations around this small-scale mixed methods analysis, additional evaluations of the core QLQ-C30 plus supplemental items should be performed to ensure that these are fit-for-purpose PRO measures in HR MDS, LB count AML, and CMML.

This study is potentially of interest to any clinical investigator working in drug development and patient-centered outcomes, as we have outlined a pragmatic approach to PRO instrument modification that includes the patient voice, as well as a strong mixed methods approach. This practice aligns with emerging best practices within the area of rare disease [4, 10]. In addition, this revised instrument may be beneficial for patients, health care practitioners, and regulatory agencies who either make or are affected by decisions regarding the treatment of HR MDS, CMML, and LB count AML. It is important to note that the items selected from the EORTC Item Library are not to be used as a single tool or new EORTC measure, but to be used in conjunction with the EORTC QLQ-C30. Further research is planned, as the EORTC QLQ-C30 and supplemental items will be tested in larger clinically-defined samples of patients with MDS, AML and CMML to evaluate their combined measurement properties in this context of use.

## Conclusion

The current study is an example of how incorporating the patient voice early in PRO instrument development and using a conceptually-driven approach to select items to increase conceptual coverage can lead to fit-for-purpose PRO instrument for clinical trials. We have used mixed methods research to select the most appropriate supplemental items from the EORTC Item Library to increase its conceptual coverage and its appropriateness for use in the specific population of patients with HR MDS, LB count AML and CMML. Ongoing psychometric evaluations in this patient population will shed further light on the appropriateness of both the original EORTC QLQ-C30 and enhanced item sets in this specific patient population.

## Abbreviations

AML: Acute myeloid leukemia
CLIN: Clinician-supported concept
CMML: Chronic myelomonocytic leukemia
EORTC: European Organization for Research and Treatment of Cancer
FAB: French American British
FDA: Food and Drug Administration
G-CSF: Granulocyte-colony stimulating factor
HR: Higher-risk
HRQOL: Health-related quality of life
IRB: Independent review board
LB: Low-blast
LIT: Literature-based concept
MDS: Myelodysplastic syndromes
PRO: Patient-reported outcome
PT: Patient-reported concept
QLQ-C30: Quality of life questionnaire-core 30 items
RAEB: Refractory anemia with excess blasts
RAEB-T: Refractory anemia with excess blasts in transformation
SD: Standard Deviation
US: United States
WHO: World Health Organization
WHOQOL: World Health Organization Quality of Life
